# Is Wortmannin-Induced Reorganization of the *trans*-Golgi Network the Key to Explain Charasome Formation?

**DOI:** 10.3389/fpls.2016.00756

**Published:** 2016-06-03

**Authors:** Ilse Foissner, Aniela Sommer, Margit Hoeftberger, Marion C. Hoepflinger, Marketa Absolonova

**Affiliations:** Department of Cell Biology/Plant Physiology, University of SalzburgSalzburg, Austria

**Keywords:** *Chara australis*, wortmannin, *trans*-Golgi network, charasome, multivesicular body, endocytosis

## Abstract

Wortmannin, a fungal metabolite and an inhibitor of phosphatidylinositol-3 (PI3) and phosphatidylinositol-4 (PI4) kinases, is widely used for the investigation and dissection of vacuolar trafficking routes and for the identification of proteins located at multivesicular bodies (MVBs). In this study, we applied wortmannin on internodal cells of the characean green alga *Chara australis*. Wortmannin was used at concentrations of 25 and 50 μM which, unlike in other cells, arrested neither constitutive, nor wounding-induced endocytosis via coated vesicles. Wortmannin caused the formation of “mixed compartments” consisting of MVBs and membranous tubules which were probably derived from the *trans*-Golgi network (TGN) and within these compartments MVBs fused into larger organelles. Most interestingly, wortmannin also caused pronounced changes in the morphology of the TGNs. After transient hypertrophy, the TGNs lost their coat and formed compact, three-dimensional meshworks of anastomosing tubules containing a central core. These meshworks had a size of up to 4 μm and a striking resemblance to charasomes, which are convoluted plasma membrane domains, and which serve to increase the area available for transporters. Our findings indicate that similar mechanisms are responsible for the formation of charasomes and the wortmannin-induced reorganization of the TGN. We hypothesize that both organelles grow because of a disturbance of clathrin-dependent membrane retrieval due to inhibition of PI3 and/or PI4 kinases. This leads to local inhibition of clathrin-mediated endocytosis during charasome formation in untreated cells and to inhibition of vesicle release from the TGN in wortmannin-treated cells, respectively. The morphological resemblance between charasomes and wortmannin-modified TGN compartments suggests that homologous proteins are involved in membrane curvature and organelle architecture.

## Introduction

Wortmannin is a furanosteroid metabolite from the fungus *Penicillium funiculosum* which is widely used as a tool for the disruption and identification of vesicular trafficking routes and for defining endosomal compartments (Robinson et al., 2008). In plant cells, wortmannin interferes with protein trafficking to the plant vacuole (daSilva et al., 2005) and it causes homotypic fusion and enlargement of multivesicular bodies (MVBs; Wang et al., 2009; Takáč et al., 2012). Wortmannin induces the fusion of vacuoles in guard cells where vacuoles are naturally fragmented after abscisic acid-induced stomata closure (Zheng et al., 2014), and on the other hand, wortmannin has been described to rescue vacuole fusion in a SNARE mutant of *Arabidopsis thaliana* (Zheng et al., 2014). In root meristems, wortmannin treatment results in the formation of abnormal vacuolar structures (Feraru et al., 2010), and in tobacco culture cells wortmannin inhibits autophagy (Takatsuka et al., 2004; Li and Vierstra, 2012). However, wortmannin also causes vacuolar cargo to be secreted to the apoplast (Pimpl et al., 2003), indicating that not only MVBs are affected, but also a compartment involved in exocytosis, e.g., the TGN (see Robinson et al., 2012). Indeed, mixed MVB/TGN compartments have been described in wortmannin-treated cells where SCAMP1, a marker of the TGN, was found to localize to the dilated, wortmannin-induced MVBs (Lam et al., 2007a). A proteomic study also confirmed the effect of wortmannin on TGNs (Takáč et al., 2012). Recently, wortmannin was found to suppress the V-ATPase activation in *A. thaliana* (Liu et al., 2016).

The huge internodes of the characean algae are useful models to study vesicular trafficking and lateral compartmentation of the plasma membrane (Foissner and Wasteneys, 2012, 2014). The cytoplasm of characean internodal cells consists of a stationary cortex in which helically oriented files of chloroplasts are anchored, and a mobile endoplasm which performs rotational streaming along actin filament bundles attached to the inner surface of the chloroplasts via interaction with myosin-coated organelles (Foissner and Wasteneys, 2014; Supplementary Figure 1). A conspicuous feature of *Chara* cells are convoluted plasma membrane domains, called charasomes. Charasomes can be stained in living cells by fluorescent plasma membrane dyes due to the increased signal caused by the superimposed plasma membrane infoldings (Schmoelzer et al., 2011; compare **Figure 6A**). Charasomes serve to accommodate a high number of H^+^-ATPases (Price and Whitecross, 1983; Schmoelzer et al., 2011), and probably also other transporters (Franceschi and Lucas, 1982; Keifer et al., 1982; Lucas et al., 1986). The H^+^-ATPases acidify the surroundings of the cell, so that the poorly membrane permeable hydrogen carbonate (${\text{HCO}}_{3}^{-}$, bicarbonate) is reduced to CO_2_ which diffuses into the cell where it is used for photosynthesis (Lucas, 1983; Price et al., 1985).

The charasomes are not evenly distributed along the cell surface and extended regions with large, numerous charasomes alternate with smaller areas containing few, small charasomes, when cells have been exposed to standard light/dark conditions (16/8 h) for at least several days (Franceschi and Lucas, 1980; Bisson et al., 1991; Schmoelzer et al., 2011). In branchlet internodal cells of *C. australis* and under steady state conditions, the distribution of charasomes correlates with the pattern of acid and alkaline regions along the surface of cells, which can be visualized by phenol red (Schmoelzer et al., 2011). However, pH bands can also develop in the absence of charasomes, and the pH banding pattern readily changes upon disturbance of the cell (Franceschi and Lucas, 1980; Bulychev et al., 2004). These newly formed pH bands are probably due to differential activation of ion pumps and/or channels, and may explain the results of other studies in which no correlation between pH bands and charasome density was found (Bisson et al., 1991).

Little is known about the formation and degradation of charasomes. Electron microscopy studies indicate that during charasome growth, vesicles derived from the TGN fuse with the plasma membrane in the absence of membrane recycling via coated vesicles (Lucas and Franceschi, 1981). The resulting tubules may again fuse with the plasma membrane and other tubules. In darkness, or in cells treated with inhibitors of photosynthesis, charasomes are degraded (e.g., Chau et al., 1994; Schmoelzer et al., 2011), probably via endocytosis. So far, it is unclear by which mechanism charasome membrane recycling is switched off or on.

The *trans*-Golgi network was first described as a “partially coated reticulum” in *Chara* internodal cells (Pesacreta and Lucas, 1984). Unlike as in many higher plant cells, the TGN of mature characean internodal cells is easy to distinguish from the Golgi body because of its distinct morphology and its location relative to the Golgi cisternae, at least in chemically fixed mature cells. Furthermore, TGN membranes are only slightly stained by zinc-iodide-osmium tetroxide in contrast to the membranes of the Golgi body (Pesacreta and Lucas, 1984). Based on these findings, the TGN was identified as an independent organelle (Pesacreta and Lucas, 1984). Later, the TGN has also been described in higher plant cells, and its identity as a separate organelle has been confirmed (e.g., Foresti and Denecke, 2008; Robinson et al., 2008 for references). The dual function of the TGN as an endocytic and secretory organelle has been proposed by localizing secretory and endocytic cargo (Viotti et al., 2010).

In an attempt to study vesicular trafficking in green algae, we treated internodal cells of *C. australis* with wortmannin. We found that wortmannin induced the fusion of MVBs with TGN-derived membranes, as described in other cells. More interestingly, wortmannin also inhibited the release of TGN vesicles, and caused a considerable reorganization of the loose TGN tubules into compact meshworks which had a stunning resemblance to charasomes. Our data indicate that similar mechanisms are involved in the formation of charasomes and wortmannin-modified TGNs, and that a similar set of proteins is involved in the development and maintenance of these complex tubular meshworks.

## Materials and methods

### Algal material, culture conditions, and inhibitor treatments

Thalli of *Chara australis* were grown in a substrate of soil, peat and sand in 10–50 L aquaria filled with distilled water. The temperature was about 20°C and fluorescent lamps provided a 16/8 h light/dark cycle. The light intensity was low (about 5 μE.m^−2^.s^−1^) in order to prevent calcification and growth of epiphytes.

For our study, we used mature, non-growing internodal cells of the branchlets of *C. australis* collected from the 3rd and the 4th upper whorl of 1–2 month-old thalli. Each whorl consisted of 5 or 6 branchlets, and each branchlet consisted of 2–3 internodal cells which varied in length between 5 and 30 mm. Prior to the experiments, the medial and distal internodal cells of the branchlets were isolated from the thallus with a small pair of scissors and incubated in artificial fresh water (10^−4^ M NaCl, 10^−4^ M KCl, 10^−3^ M CaCl_2_) for at least 1 day. Cells were then incubated in wortmannin-containing AFW, or in the appropriate DMSO-containing solution (control).

Wortmannin (Enzo Life Sciences, Lausen, Switzerland) was dissolved in dimethyl sulfoxide (DMSO) at a concentration of 5 mM and diluted with AFW. During the course of this study we used 25 and 50 μM working solutions. Controls contained the corresponding amounts of the solvent (up to 1%). For studying the internalization of FM/AM dyes, we additionally tested wortmannin solutions diluted from a 10 mM stock solution in order to decrease the DMSO content (maximal 0.5%) which could eventually lead to non-endocytotic uptake of the styryl dyes. The pH of the artificial fresh water was not significantly altered by the addition of DMSO or wortmannin.

The velocity of cytoplasmic streaming was measured in the light microscope using a 40x objective lens and a stop watch. The movement of at least two structures (organelles) in different regions of the bulk streaming mass was analyzed per cell and the highest value was used for statistical analysis.

### pH banding and *in vivo* staining

The pH banding pattern of internodal cells was documented using phenol red (phenolsulfonphthalein; Sigma, St. Louis, USA) at a concentration of 4 μM dissolved in AFW from a stock solution of 10 mM in distilled water.

For *in vivo* staining of charasomes and endosomes internodal cells were pulse labeled for 5 min with green fluorescent FM1-43FX (N-(3-triethylammoniumpropyl)-4-(4-(dibutylamino)styryl)pyridinium dibromide) (Invitrogen, Carlsbad, USA) and AM1-44 (Biotium, Hayward, USA), or with red fluorescent FM4-64 (N-(3-triethylammoniumpropyl)-4-(6-(4-(diethylamino)phenyl)hexatrienyl)pyridinium dibromide) (Invitrogen) and AM4-65 (Biotium). All dyes were used at a concentration of 10 μM.

**Figure 1:**
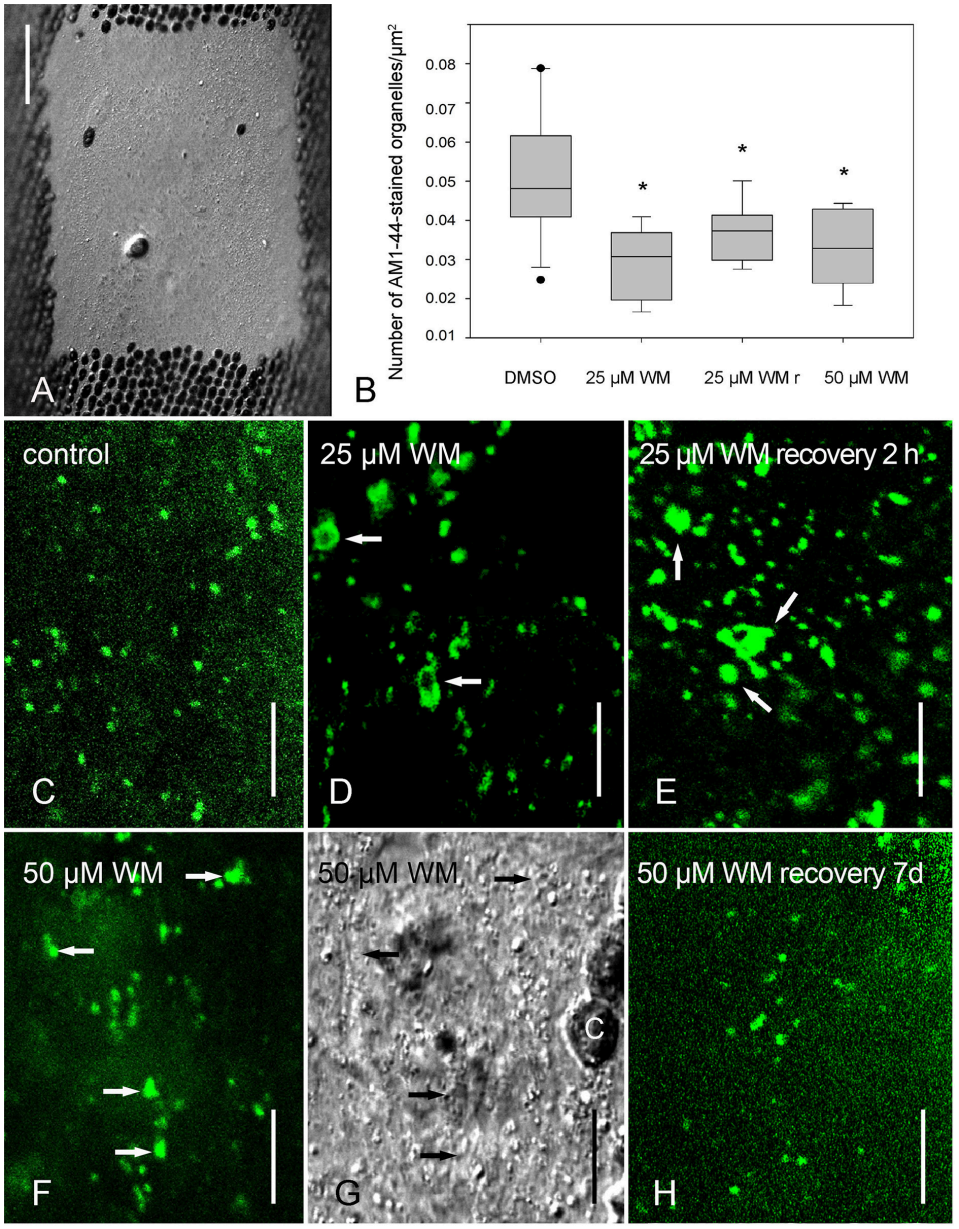
**Effect of wortmannin on internalization of AM1-44 in internodal cells of ***Chara australis***. (A)** Chloroplast-free window for imaging endosomal organelles (DIC) **(B)** Relative numbers of AM1-44-stained organelles in control cells, in cells treated with 25 μM wortmannin (WM) for 2.5 h, in cells recovering from this treatment for 2 h in artificial fresh water (WMr) and in cells treated with 50 μM wortmannin for 2.5 h are compared in box-and-whisker plots. Shown are median values with upper and lower quartiles (boxes), whiskers indicating the 10th and 90th percentiles, and outliers (dots) with *n* = between 5 and 17. Differences between median values of control and treated cells are significant (asterisks; one way analysis of variance, *P* = 0.001). **(C**–**H)** AM1-44-stained organelles in cells treated with 0.5% DMSO **(C)**, with 25 μM wortmannin for 2.5 h **(D)**, with 25 μM wortmannin for 2.5 h followed by 2 h “recovery” in artificial fresh water **(E)**, with 50 μM wortmannin for 2.5 h (**F**,**G** is the corresponding DIC image), and with 50 μM wortmannin for 2.5 h, followed by 7 d recovery in artificial fresh water (**H**, compare with **F**). Arrows indicate enlarged organelles in response to wortmannin treatment; C, chloroplast. Bars are 50 μm **(A)** and 10 μm **(C–H)**.

The *in vivo* detection and counting of endosomal organelles stained by styryl dyes was hampered by the strong autofluorescence of the compact chloroplast files. Therefore, we applied the “window technique,” which is generally used for light microscopical observation of the endoplasm in characean cells (Kamitsubo, 1972). This method consists in local strong illumination of the cells which causes chloroplasts to bleach, swell and eventually detach from the irradiated area. *Chara* cells used in our experiments were locally irradiated with the blue light of a halide microscope lamp for 3 min at least 1 day prior to the experiments, which resulted in a chloroplast-free window with an area of about 30,000 μm^2^ (ca. 200 × 150 μm wide rectangle; compare Figure 1A). Data were collected from individual images and care was taken to analyze non-overlapping regions of endoplasm. Fluorescent particles were counted and analyzed using ImageJ (http://imagej.nih.gov/ij).

### Immunofluorescence

Fixation and staining protocols for indirect immunofluorescence were as described by Schmoelzer et al. (2011). Primary antibodies used for this study were rabbit polyclonal anti-OsSCAMP1 (Lam et al., 2007a; generously provided by Liwen Jiang, University of Hongkong) used at a concentration of 40 μg per ml and rabbit polyclonal anti-CaARA7 which was used at a concentration of 3.6 μg per ml (Hoepflinger et al., 2015). Secondary antibodies were anti-rabbit IgG Alexa 488 (Invitrogen) diluted 2:1000, or anti rabbit IgG Alexa Fluor 546 (Invitrogen) diluted 3:1000. All antibodies were diluted in blocking buffer [1% (w/v) bovine serum albumin and 50 mM glycine in phosphate buffered saline (PBS), pH 7.2].

For double labeling experiments, we used basically the same fixing and staining protocol as above with some modifications because both primary antibodies were raised in rabbits. After incubation with the first primary antibody (rabbit polyclonal anti-CaARA7), samples were incubated with a monovalent anti-rabbit Fab' fragment coupled to Alexa 488 (Nanoprobes, USA), then washed with PBS and subsequently post-fixed for 30 min in 1% (v/v) glutaraldehyde in PBS. Samples were rinsed overnight with PBS buffer and then blocked with 1% (w/v) bovine serum albumin and 50 mM glycine in PBS, followed by the incubation with the second primary antibody anti-OsSCAMP1. All subsequent steps were as described in Schmoelzer et al. (2011).

### Confocal laser scanning microscopy and statistical analysis

The confocal laser scanning microscopes used in this study were a Leica (Mannheim, Germany) TCS SP5 coupled to a DMI 6000B inverted microscope, and a Zeiss (Jena, Germany) LSM 510 coupled to a Zeiss Axiovert inverted microscope. For the excitation of Alexa 488, FM1-43, and AM1-44, we used the 488 nm line of the argon laser, and the emitted fluorescence was detected in the range 505–550 nm. For the excitation of the red fluorescent dyes FM4-64 and AM4-65, we used the 514 nm line of the argon laser and the detection of the fluorescent signal was between 660 and 720 nm. Alexa Fluor 546 was excited with the 561 nm line of a diode pumped solid state laser, and detected in the range 580–620 nm. For double-stained samples, we always used the sequential scanning mode. All images included in this study are single optical sections with a thickness of about 1.2 μm, and are positioned with vertical sides parallel to the long axes of the cells. Images were taken using a 40x water immersion objective with a numerical aperture of 1.2, or a 63x water immersion objective with a numerical aperture of 1.4. Statistical analysis of charasome area fraction and abundance of endocytic vesicles was performed using ImageJ and SigmaPlot (Systat Software, San Jose, USA). All experiments were repeated at least twice.

### Electron microscopy

Chemical fixation of branchlet internodal cells of *C. australis* was as described in Foissner (1991). Briefly, cells were fixed for 30 min at room temperature in 1% glutaraldehyde dissolved in phosphate buffer, pH 6.8. Following several washes in buffer, cells were postfixed overnight at 4°C in 2% OsO_4_ dissolved in buffer. After dehydration in an ethanol series at 4°C, cells were embedded in Agar low viscosity resin (Agar Scientific, Essex, Great Britain). After staining with uranyl acetate and lead citrate, micrographs of ultrathin section were taken at elastic bright-field mode with a LEO 912 transmission electron microscope equipped with in-column energy filter (Zeiss, Oberkochen, Germany).

## Results

### Wortmannin does not arrest endocytosis in characean internodal cells

In order to get a first impression how wortmannin affected the branchlet internodal cells, we measured the rate of cytoplasmic streaming, which depends on the interaction of myosin-coated organelles with subcortical actin bundles attached to the inner side of the stationary chloroplasts (Shimmen and Yokota, 2004 for review). We found that all concentrations and treatment times which had an effect on the fine structure of cells, also significantly decreased the rate of cytoplasmic streaming. The extent of streaming inhibition varied between cultures, and depended on the time period elapsing between isolation of individual cells and the beginning of the experiment. In general, cells from older cultures (2 months) were less affected by wortmannin than cells from young cultures (≤ 1 month), and cells which had been isolated for a longer time period were more resistant to the effect of wortmannin than cells freshly isolated from the thallus. Since cytoplasmic streaming is vital for most cellular processes, for further experiments we only used cells in which the streaming rate was above 70% of the control rate as shown in Table 1. Table 1 also shows that 25 and 50 μM wortmannin significantly decreased the number of pH bands per cell which were visualized by incubating cells in phenol red, a pH indicating dye. The pH banding activity depends on photosynthesis and on cytoplasmic streaming (Bulychev et al., 2001). The wortmannin-induced reduction in pH banding activity was therefore probably due to the lower streaming velocity, although we presently cannot exclude a possible effect on the rate of photosynthesis.

**Table 1:** **Effect of wortmannin on the velocity of cytoplasmic streaming and on the pH banding activity of branchlet internodal cells of ***Chara australis*****.

	**DMSO 0.5%**	**25 μM Wortmannin**	**50 μM Wortmannin**
v Streaming (μm.s^−1^)	73.7 ± 6.6 (15)	60.8 ± 11.7 (13)^*^	53.6 ± 4.3 (15)^*^
v Streaming (μm.s^−1^), recovery 4 d	79.9 ± 4.8 (15)	57.8 ± 9.8 (13)^*^	62.7 ± 9.5 (13)^*^
v Streaming (μm.s^−1^), recovery 7 d	74.7 ± 5.1 (15)	68.6 ± 11.9 (13)^+^	67.7 ± 10.6 (13)^+^
pH bands/cell	1.3 ± 1.0 (15)	0 ± 0 (13)^*^	0 ± 0 (13)^*^
pH bands/cell; recovery 4 d	1.1 ± 0.3 (15)	0.1 ± 0.3 (13)^*^	0.1 ± 0.3 (13)^*^
pH bands/cell; recovery 7 d	0.9 ± 0.4 (15)	0.9 ± 0.6 (13)	0.6 ± 0.5 (13)^+^

Cells were treated with solutions containing wortmannin or DMSO and supplemented with 4 μM phenol red. After 2 h treatment under room light, the number of alkaline bands was counted, and the velocity of streaming was measured. Following treatment, cells were allowed to recover in artificial fresh water supplemented with 4 μM phenol red. Data are means ± SD (number of cells). Significant differences between controls and treatments were calculated according to Students t-test and are indicated by

* P ≤ 0.01 and

+ *P ≤ 0.05*.

In a variety of cells, wortmannin has been reported to inhibit endocytosis probably via alterations of clathrin-coated domains (Ito et al., 2012 and references therein). We therefore investigated the effect of wortmannin on the internalization of styryl dyes, established markers for the plasma membrane and for organelles involved in endocytosis (Griffing, 2008). Cells were pretreated with solvent (control) or wortmannin for 2.5 h, and pulse labeled with AM4-65. After 30 min incubation in dye-free control, or wortmannin solution, time series were taken. Supplementary Video 1 shows AM4-65 stained charasomes in the cortex of a control cell. Between the stationary charasomes AM4-65-fluorescent, fast moving structures with a diameter of < 250 nm (at the resolution limit) were occasionally seen (compare Klima and Foissner, 2008). To our surprise, such small mobile organelles, putative endosomes, were also present in wortmannin-treated cells (Supplementary Video 2). The cortex of wortmannin-treated cells additionally contained larger, mobile organelles which had a diameter of up to 700 nm. They performed saltatory movements along the plasma membrane, which alternated with periods of immobility (Supplementary Video 2). In order to quantify FM/AM internalization, a chloroplast free window was produced at least 1 day prior to the experiments, to get an undisturbed view into the endoplasm and to count the number of fluorescent organelles (Figures 1A,C–H). The endoplasm as well contained abundant FM/AM-fluorescent organelles, although their number was significantly lower than in solvent-treated cells (Figure 1B). The maximum size of fluorescent endoplasmic organelles in control internodes (Figure 1C) was 2.9 μm (*n* = 625), whereas in cells treated with wortmannin, enlarged organelles, including ring-like “wortmannin compartments,” and clusters of organelles with diameters of up to 4.2 μm (*n* = 371) were observed (Figures 1D,F). This suggests that the reduction in organelle number was at least partly due to the formation of wortmannin-induced compartments. With respect to size and number of fluorescent structures detected in wortmannin-treated cells, we could not see a concentration-dependent effect, namely there were no significant differences between 25 and 50 μM. Size and number of fluorescent organelles in cells treated with 10 μM wortmannin or less were not significantly different from those in control cells.

Largest AM1-44-stained particles with a size of up to 8 μm were observed in cells “recovering” in artificial fresh water from a 30 min to 2.5 h treatment with 25 μM wortmannin (Figure 1E). These data indicate that a “recovery” up to 2 h in fact reflects a prolonged treatment time because of the tight binding of wortmannin to its target enzyme(s) (see Section Discussion). Wortmannin-induced compartments were also observed when cells were first pulse-labeled with FM/AM dyes and subsequently incubated in wortmannin (not shown). The effects of wortmannin on cytoplasmic streaming and pH banding activity were reversible when cells were allowed to recover (Table 1). It took, however, several days in artificial fresh water until nearly normal streaming velocities and pH banding activities were regained. After 1 week recovery from wortmannin treatment (25 and 50 μM), also the size of FM/AM-stained organelles was similar to that in control cells (Figure 1H).

Size, abundance and distribution of charasomes were not affected by wortmannin up to a concentration of 50 μM and a treatment time of up to 3 h (data not shown).

### Wortmannin induces clustering and enlargement of organelles recognized by markers for MVBs and TGNs

**Figure 2:**
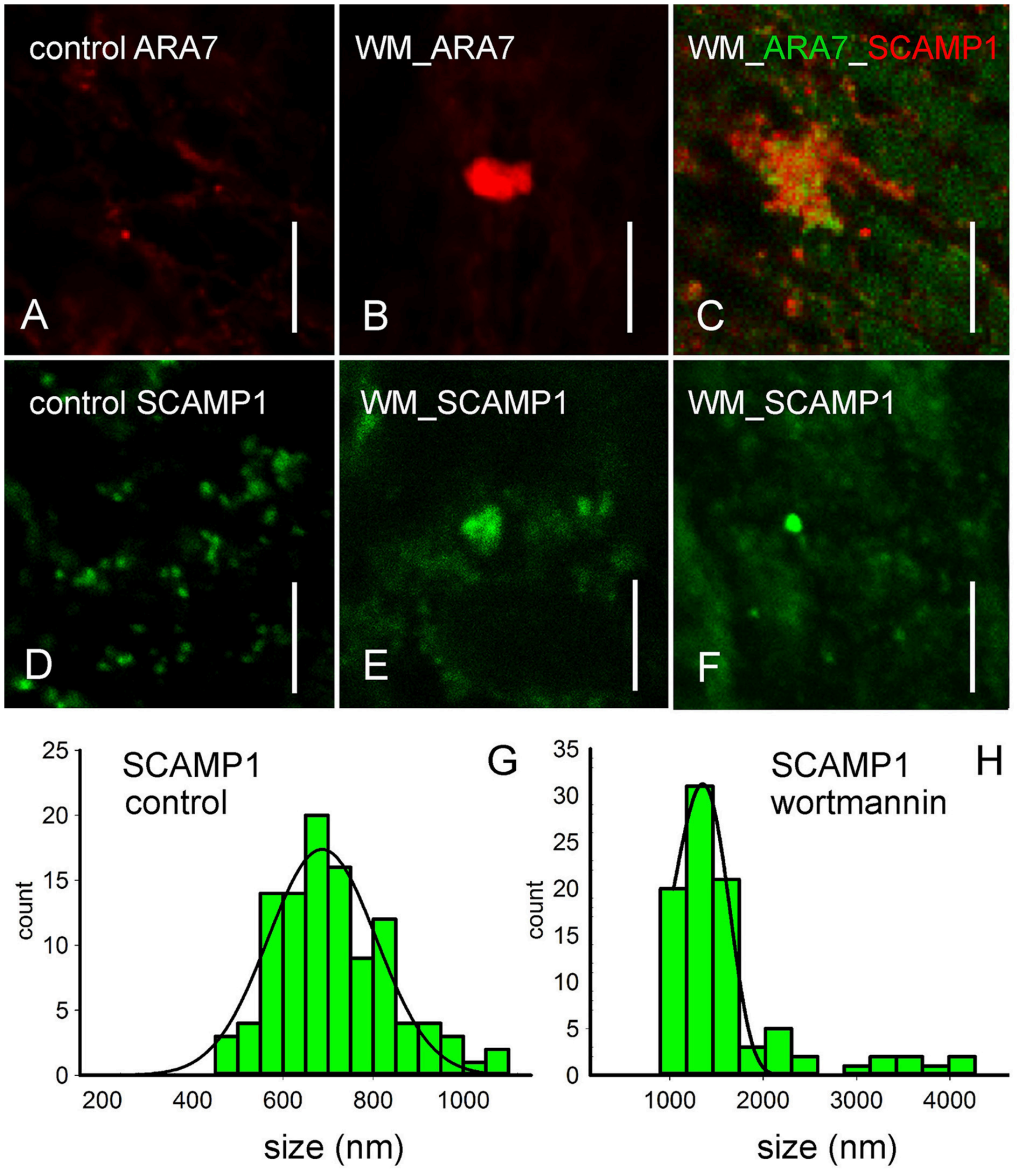
**Immunofluorescence with antibodies against endosomal markers of control cells (A,D), of internodal cells treated with 50 μM wortmannin for 2 h (B,C,E,F) and corresponding histograms (G,H)**. **(A,B)** Immunofluorescence with an antibody against CaARA7, a marker for MVBs, shows small punctate organelles in untreated cells and larger compartments **(B)** in wortmannin-treated cells. **(C)** Double immunofluorescence with anti-CaARA7 and anti-OsSCAMP1, a marker of TGNs suggests that groups of small MVBs intermingle with TGN membranes. **(D–F)** Immunofluorescence with anti-OsSCAMP1. The antibody recognizes abundant small organelles with various shapes in the endoplasm of an untreated cell **(D)**. In wortmannin-treated cells, large SCAMP1-positive aggregates **(E)** and large, globular organelles **(F)** are present. **(G,H)** The histograms show the size distributions of SCAMP1-positive organelles in solvent-treated cells, and in cells treated with 50 μM wortmannin for 2 h. Bars are 10 μm.

**Figure 3:**
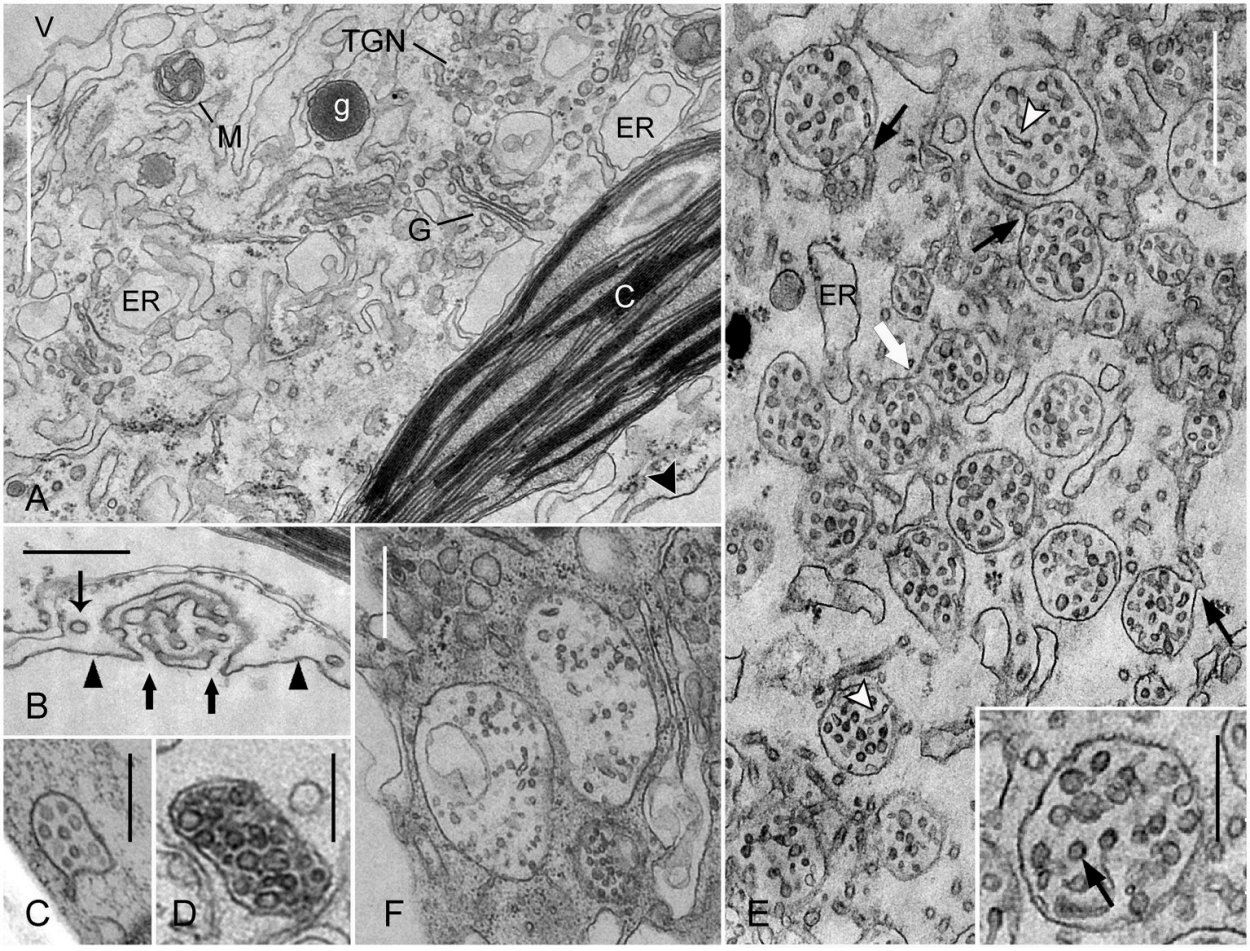
**Effect of wortmannin on MVBs in ***Chara*** internodal cells**. Cells were treated with 50 μM wortmannin for 2 h **(A,B,E)**, and with 25 μM for 30 min followed by 2 h recovery **(F)**. Images of multivesicular bodies **(C,D)** are from untreated cells. **(A)** Cross-section through the chloroplast (C)-containing cortex near the plasma membrane (arrow head), and the endoplasm located between cortex and vacuole (V). Wortmannin does not affect the fine structure of chloroplasts, Golgi bodies (G), glycosomes (g), mitochondria (M), and endoplasmic reticulum (ER). Note also that the TGN visible in this area has a normal shape and morphology. **(B)** The charasome in a wortmannin-treated cell is similar to those in control cells. Thick arrows indicate the openings of charasome tubules to the cell wall space. The thin arrow points to a coated vesicle, probably released from the smooth plasma membrane (arrow heads). **(C,D)** MVBs in untreated branchlet internodal cells are small and variable in shape. **(E)** In wortmannin-treated cells, MVBs form large clusters with intertwining membrane tubules. The white arrow indicates fusion between two MVBs, and the black arrows point to continuities between MVBs and membrane tubules. Arrow heads indicate tubules within MVBs. The arrow in the inset points to a cross-sectioned tubule or vesicle with a central core. **(F)** Large MVBs in a cell recovering from wortmannin-treatment. Bars are 1 μm **(A)**, 500 nm **(E,B,F)**, and 250 nm (**C,D** and inset in **E**).

In order to identify the nature of wortmannin compartments, we applied immunofluorescence using antibodies against CaARA7 and OsSCAMP1 which are confident markers for MVBs and TGNs, respectively (Lee et al., 2004; Ueda et al., 2004; Lam et al., 2007a; Hoepflinger et al., 2015). In solvent treated control cells, the antibody against ARA7 labeled organelles (MVBs) with a size of up to 1.0 μm (Figure 2A). Cells treated with 50 μM wortmannin additionally contained up to 3.5 μm large compartments (Figures 2B,C; see Hoepflinger et al., 2015 for size histograms). Organelles recognized by anti-OsSCAMP1 in control cells had a roundish shape and a size between 0.5 and 1.1 μm (Figures 2D,G), corresponding to the shape and size of TGNs seen on electron micrographs (e.g., Figure 3A). In wortmannin-treated cells the antibody against SCAMP1 labeled aggregates of smaller organelles which had a size of up to 4.3 μm or large, globular organelles with a diameter of up to 2.6 μm (Figures 2E,F,H). Immunolabeling of wortmannin-treated cells with both antibodies revealed that all large clusters carrying the ARA7 epitope were additionally recognized by anti-OsSCAMP1 although the fluorescent signals did not precisely overlap (Figure 2C). Unlike as in higher plant cells (Lam et al., 2008), the plasma membrane was not recognized by anti-OsSCAMP1. Secondary antibodies alone gave no staining (not shown).

### Accumulation and fusion of MVBs in *Chara* internodal cells involves participation of the TGN

**Figure 4:**
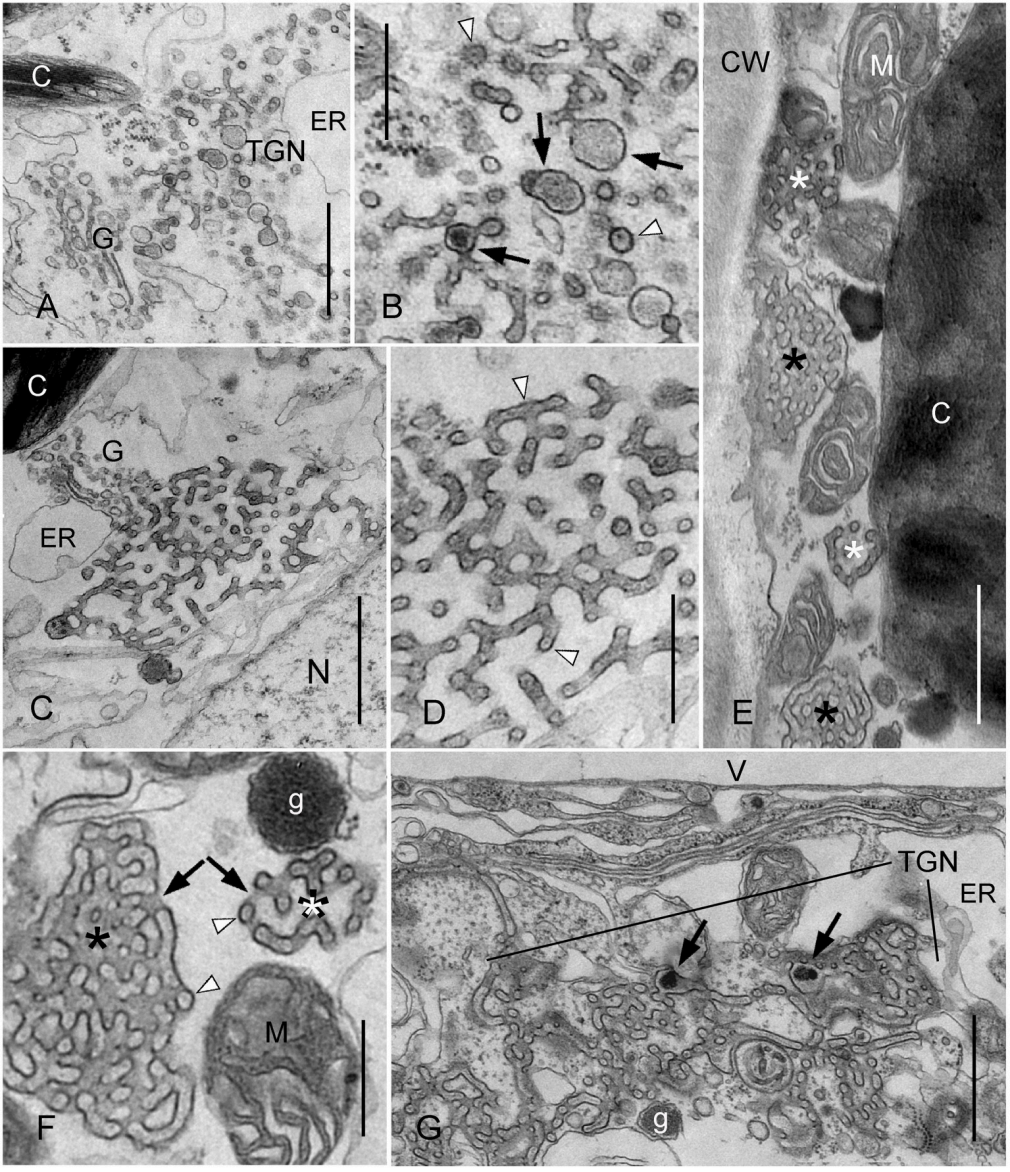
**Effect of wortmannin on TGNs in ***Chara*** internodal cells**. Cells were treated with 50 μM wortmannin for 2 h **(A–F)**, and with 25 μM for 30 min, followed by a recovery for 2 h **(G)**. **(A,B)** Enlarged TGN near a Golgi body (G) in a wortmannin-treated cell (C, chloroplast; ER, endoplasmic reticulum). The higher magnification in **(B)** shows smooth and coated tubules, and vesicles which occasionally contain a central core (arrow heads). Enlarged regions with amorphous content are indicated by arrows. **(C,D)** Wortmannin-modified TGN located in the endoplasm between a Golgi body (G) and a nucleus (N). Arrow heads in the enlarged detail **(D)** indicate tubules with cross- or longitudinal sectioned central core. Note uniform diameter of tubules and absence of coated regions. **(E)** Charasomes (black asterisks), mitochondria (M), and wortmannin-modified TGNs (white asterisks) squeezed between cell wall (CW) and chloroplast **(C)**. **(F)** Higher magnification of a charasome (black asterisk) and a wortmannin-modified compact TGN (white asterisk) in the cortex. Arrows indicate openings of the charasome and the TGN to the cytoplasmic space, respectively. Arrow heads indicate tubules or vesicles with central core. g, glycosome; M, mitochondrion. **(G)** Huge TGN complex in a cell recovering from wortmannin-treatment. g, glycosome; V, vacuole. Arrows point to enlarged areas with electron dense granular material. Bars are 1 μm **(A,C,E,G)** and 500 nm **(B,D,F)**.

To further verify the results obtained by immunolabeling, cells were fixed and processed for electron microscopy. The effect of wortmannin was investigated in cells exposed to 25 μM wortmannin for 30 min, in cells treated with 50 μM wortmannin for 2 h, and in cells recovering for 2 h from a 30 min treatment with 25 μM wortmannin. Because of the large size of the mature internodes, only chemical fixation could be applied. Wortmannin had no detectable effect on the fine structure of cortical chloroplasts and plasma membrane (Figures 3A,B). Smooth plasma membrane regions alternated with charasomes, where the plasma membrane formed complex labyrinths of anastomosing tubules (Figure 3B; Lucas and Franceschi, 1981; Chau et al., 1994). As in controls, coated pits (not shown) and coated vesicles were present at the smooth plasma membrane regions (Figure 3B), and less frequent at mature charasomes (compare Sommer et al., 2015). In the endoplasm, wortmannin modified the fine structure and distribution of MVBs and TGNs (see below) but the fine structure of other organelles like Golgi bodies, mitochondria (Figure 3A), nuclei (Figure 4C), and peroxisomes (not shown) were not affected. Just as in control cells, different kinds of vesicles were present between the numerous cisternae of the endoplasmic reticulum (Figure 3A). These observations indicate that wortmannin very specifically targeted the TGNs and the MVBs in *Chara* internodal cells.

The MVBs in the untreated, chemically fixed, mature branchlet internodal cells investigated in this study mostly had an irregular, flattened, or elongate shape and a mean length of 0.6 ± 0.2 μm SD (*n* = 24; Figures 3C,D). Membranous tubules were occasionally seen to extend from their outer membrane (Figure 3C). Morphology and size of MVBs were similar in cells treated with 25 μM wortmannin for 30 min, but aggregates of MVBs were sometimes observed (not shown). Treatment with 50 μM wortmannin for 2 h had a stronger impact on distribution, shape, and morphology of these organelles. The cells contained huge clusters of roundish MVBs, which were surrounded by and continuous with membranous tubules (Figure 3E). The mean diameter of these MVBs (0.5 ± 0.1 μm; *n* = 39) was similar to the size of MVBs in untreated cells (see above), but due to their roundish shape a higher volume can be assumed. Inside these clusters, MVBs fused into larger organelles (white arrow in Figure 3E). The MVBs in wortmannin-treated cells contained abundant tubules (Figure 3E), in contrast to the MVBs in control cells which predominantly contained vesicles (Figures 3C,D). The diameter of the tubules between and inside the MVBs and the occasional presence of a central core (Figure 3E, inset), a typical structure of TGNs in *Chara* internodal cells (see below), suggest that these tubules were of TGN origin. Taken together, these data indicate that wortmannin-induced MVB clusters correspond to “mixed compartments.” This is consistent with our results of double immunofluorescence using anti-ARA7, a marker for MVBs, and anti-SCAMP1, a marker for TGNs (Lam et al., 2007a). Inside these clusters, MVBs fused with each other and with tubular membranes derived from TGNs. In contrast to TGNs, no single MVBs were observed outside the wortmannin-induced clusters.

Interestingly, the largest MVBs with a diameter of up to 1.2 μm were found in cells “recovering” for 2 h in artificial fresh water from 30 min treatment with 25 μM wortmannin (Figure 3F). Their MVBs had a roundish, cup-, or doughnut-like shape and their mean size (diameter; 0.8 ± 0.2 SD, *n* = 24) was significantly larger than the length of MVBs in control cells (*P* ≤ 0.0001, Students *t*-test). These large MVBs were no longer surrounded by and continuous with membranous tubules, suggesting that these got used up during fusion.

### Wortmannin causes reorganization of the TGNs into charasome-like structures

In addition to the effect of wortmannin on MVBs, we found that this substance also profoundly altered the morphology of TGNs in *Chara* internodal cells. Interestingly, wortmannin did not affect all TGNs of a cell equally. TGNs with normal size and morphology, as well as TGN-derived glycosomes (polysaccharide-containing vesicles; (Franceschi and Lucas, 1981b); Figure 3A) were also present in the endoplasm of cells with a high number of compact wortmannin-modified TGNs (see below).

Figures 4A,B show a detail of a cell treated with 50 μM wortmannin for 2 h. The TGN visible in this area was enlarged, but its fine structure was similar to that in untreated cells. It consisted of a loose meshwork of smooth and coated tubules, which was often laterally associated with the Golgi bodies (Pesacreta and Lucas, 1984). The tubules alternated with enlarged regions which contained granular or amorphous material, and which probably gave rise to glycosomes (Pesacreta and Lucas, 1984). Coated and non-coated vesicles were present at the periphery of the TGN. Occasionally, a central core with a diameter of 8–10 nm was seen within tubules or vesicles (Figure 4B).

A much stronger effect of wortmannin on the structure of TGNs is seen in Figures 4C,D. These organelles consisted of smooth tubules with a more uniform diameter, and neither coated vesicles, nor vesicles with a smooth membrane were seen at their periphery. However, even such strongly modified TGNs were still associated with Golgi bodies (Figure 4C). The most severe effect of wortmannin on TGNs is illustrated in Figures 4E,F. These TGNs consisted of smooth tubules, as described above, but formed a more roundish, compact organelle in which the distance between tubules was similar to the diameter of the tubules. All tubules contained a central core, which either had the shape of a filament, or appeared like a row of dots (Figures 4D,F). In control cells, TGNs were predominantly located in the streaming endoplasm. Many of the compact wortmannin-modified TGNs, however, were found in the cortex (Figures 4E,F), and most probably corresponded to the mobile FM/AM-stained organelles visible in Supplementary Video 2. It is feasible that these compact TGNs move from the endoplasm toward the plasma membrane along actin filament bundles extending through the chloroplast layer (Foissner and Wasteneys, 2014), but we can also not exclude the possibility that these TGNs form *de novo* in the cortex.

Most interestingly, we found that the wortmannin-induced compact TGNs had a striking resemblance with charasomes. Electron microscopical images of both organelles typically showed three radiating tubules, and in addition, one or two arms extending toward regions outside the section (Figure 4F; compare Lucas and Smith, 1976; Franceschi and Lucas, 1980). The tubules were interconnected and formed a complex, three-dimensional structure. The diameter of charasome tubules described in previous studies varied between 20 and 40 nm (Franceschi and Lucas, 1980; Lucas et al., 1986; Chau et al., 1994), and is obviously dependent on fixation and embedding protocol. The mean diameter of the tubules in our preparations measured at their narrowest region, was 69.8 ± 5.7 nm in charasomes, and 67.7 ± 7.6 nm in wortmannin-modified TGNs (no significant difference; 8 organelles from 3 different cells were measured). The charasome tubules, however, were less straight, and their diameter was not as uniform as the diameter of TGN tubules, because they were thicker at their base, i.e., at the site at which the tubules branched or anastomosed, respectively, at least after chemical fixation as used in our study. The tubules in wortmannin-modified TGNs contained a central core surrounded by electron lucent space (Figures 4D,F). Such a core is also present in the charasome tubules, although it is less distinct (Figure 4F). Both charasomes and wortmannin-modified TGNs are open to the surrounding cytoplasm, and the space between the tubules is thus cytosolic (arrows in Figures 4F,G). The cytosolic space between the charasome tubules often appears darker than that of compact TGNs, because it contains additional membrane material from uneven background tubules (Lucas and Franceschi, 1981). On the other hand, the lumen of wortmannin-modified TGN tubules appears slightly darker than those of the charasome tubules. As a result, images of the compact wortmannin-modified TGN complexes appear nearly as a negative of the images of the charasomes, when fixed and stained under identical conditions. The most important difference between charasomes and wortmannin-modified TGNs is, that the lumen of charasome tubules is open to the cell wall space or periplasm (Figure 3B; Franceschi and Lucas, 1980), whereas wortmannin-modified TGN tubules seemed to be always closed, even when found near the plasma membrane (Figures 4F,E).

We also investigated cells which recovered from a 30 min treatment with 25 μM wortmannin. After 2 h in artificial fresh water, these cells contained larger FM/AM-stained compartments than before recovery (see above). The compartments were also larger than those observed in cells treated with 50 μM wortmannin for 2 h. The large compartments corresponded to huge clews of non-coated, loosely arranged TGN tubules (Figure 4G). All tubules contained a central core, but in addition, electron dense amorphous material and (clathrin-) coated pits (not shown) were present in some wider regions. They might represent TGN areas which were able to regenerate at least partly, or remnants of enlarged TGNs similar to that shown in Figure 4C. Compact charasome-like TGNs were also present although in lower numbers than in cells treated with 50 μM wortmannin for 2 h.

### Wortmannin does not impair wound healing

We then wanted to know whether wortmannin interfered with the healing of wounds induced by puncturing and by UV irradiation, since the process of cell wall repair involves secretion as well as endocytic events. The two types of wound healing differed in the extent of plasma membrane recycling, and in the composition of the wound wall deposited, as described earlier (Foissner and Wasteneys, 2012). Before wounding, cells were treated with 50 μM wortmannin for 2 h. After injuring the cell wall by puncturing with tungsten needles or by local irradiation with intense UV light (Klima and Foissner, 2011; Foissner and Wasteneys, 2012), cells were left in the wortmannin solution for 1 day. Control cells were wounded and recovered in AFW containing the adequate amount of solvent.

**Figure 5:**
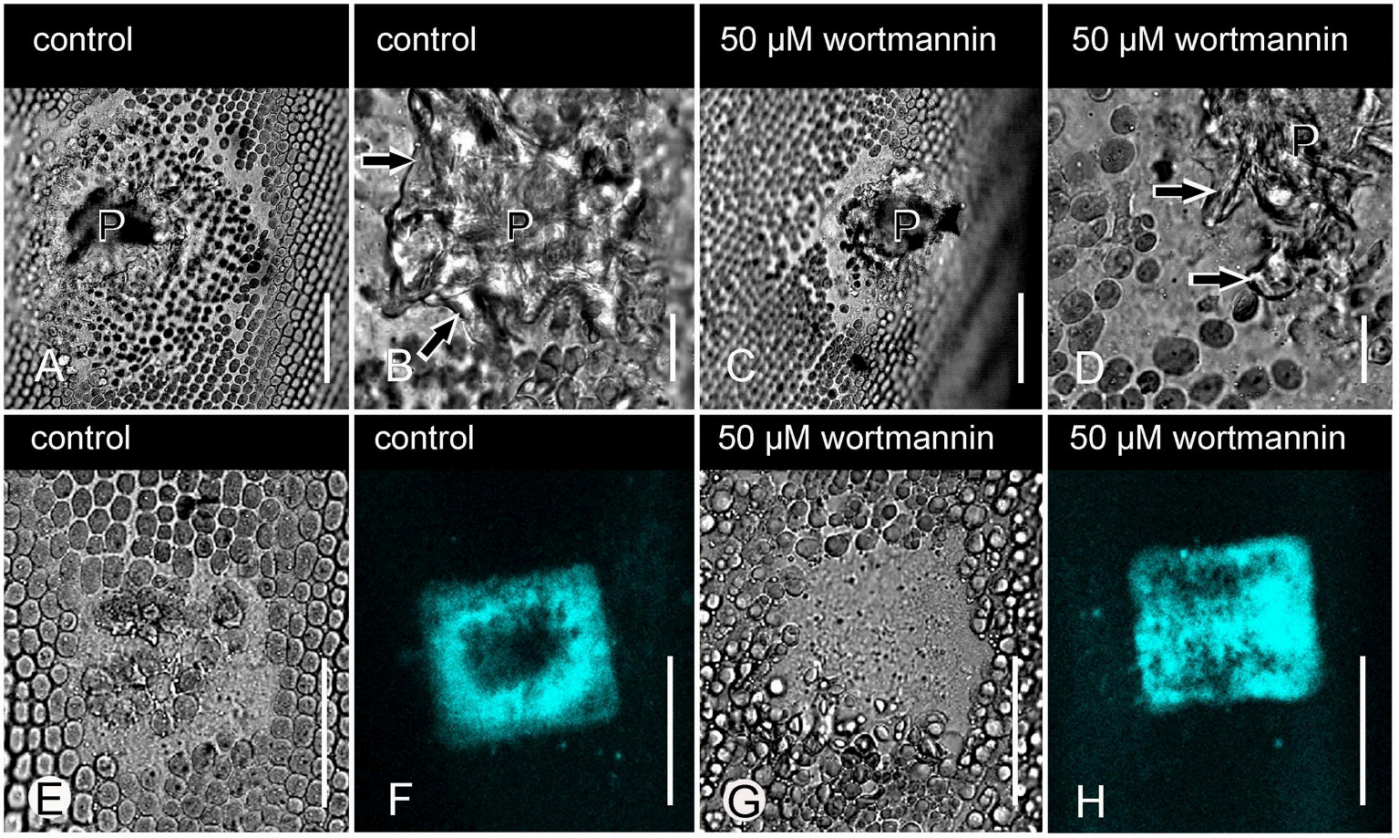
**Effect of wortmannin on wound response in ***Chara*** internodal cells**. **(A–D)** DIC images of healed puncture wounds in a DMSO-treated cell (control; **A,B**), and in a cell treated with 50 μM wortmannin **(C,D)**. The wound plug (P), consisting of vacuolar inclusions, is covered by a cellulosic wound wall (arrows in **B**,**D)**. **(E–H)** Healed UV-induced wounds in a control cell **(E,F)**, and in a cell treated with 50 μM wortmannin before and after wounding **(G,H)**. **(E,G)** are DIC images and **(F,H)** are the corresponding images of callose visualized after staining with sirofluor. Bars are 50 μm **(A,C,E–H)** and 10 μm **(B,D)**.

Puncturing internodal cells locally removed cortical chloroplasts, and after withdrawal of the needle, the cell wall hole became sealed by vacuolar inclusions (Figure 5A). Onto this wound plug, a cellulose-containing wound wall was deposited by fusion of wall forming vesicles and recycling of excess plasma membrane via coated vesicles (Figure 5B; Foissner and Wasteneys, 2012). A wound wall was also deposited onto the surrounding, damaged cortical region, and the diameter of this area was a suitable criterion for the efficacy of wound healing. Figures 5C,D show that the shape and morphology of healed puncture wounds in a wortmannin-treated cell were similar to those in a control cell, and the diameters of damaged cortical areas were not significantly different (Table 2). The survival rate was 100% in control cells and 90% in wortmannin-treated cells.

**Table 2:** **Effect of wortmannin on wound healing in branchlet internodal cells of ***Chara australis*****.

	**DMSO 0.5%**	**50 μM Wortmannin**	***P***
Puncture wounds (diameter of injured cortical area in μm)	153 ± 58 (13)	154 ± 55 (10)	0.9
UV-induced wounds (thickness of callose wound wall in μm)	3.6 ± 1.3 (7)	4.1 ± 1.8 (7)	0.6

*Before wounding, cells were treated with 50 μM wortmannin or DMSO for 2 h. After injury, cells were left in the wortmannin or DMSO solution for 1 day. Data are means ± SD (number of cells). Differences between controls and treatments were calculated according to Students t-test and were not significant*.

Local irradiation of the chloroplast-containing cell cortex with intense light caused the local accumulation of secretory vesicles (glycosomes) and FM/AM-stained putative endosomes between plasma membrane and chloroplasts (Klima and Foissner, 2011; Foissner and Wasteneys, 2012). Glycosomes and endosomes fuse with the plasma membrane and with each other in the absence of membrane recycling, and callose is the characteristic polysaccharide of such wounds. Under the experimental conditions used during this investigation, AM1-44-stained organelles appeared in the cortex within 6 min, both in control and in cells treated with 25 (not shown) or 50 μM wortmannin. All injured cells survived until the following day and produced similar amounts of callose (Figures 5E–H; Table 2).

### Effect of wortmannin on charasome degradation and charasome formation

Charasomes form in the light and are degraded upon dark-incubation (Schmoelzer et al., 2011 and references therein). Formation requires fusion of TGN vesicles with the plasma membrane and local inhibition of endocytosis (Lucas and Franceschi, 1981; Franceschi and Lucas, 1982). Charasome degradation has not been studied so far, but most likely involves membrane recycling via coated vesicles. We were therefore interested to know whether wortmannin had an effect on light-induced charasome formation and darkness-induced charasome degradation.

**Figure 6:**
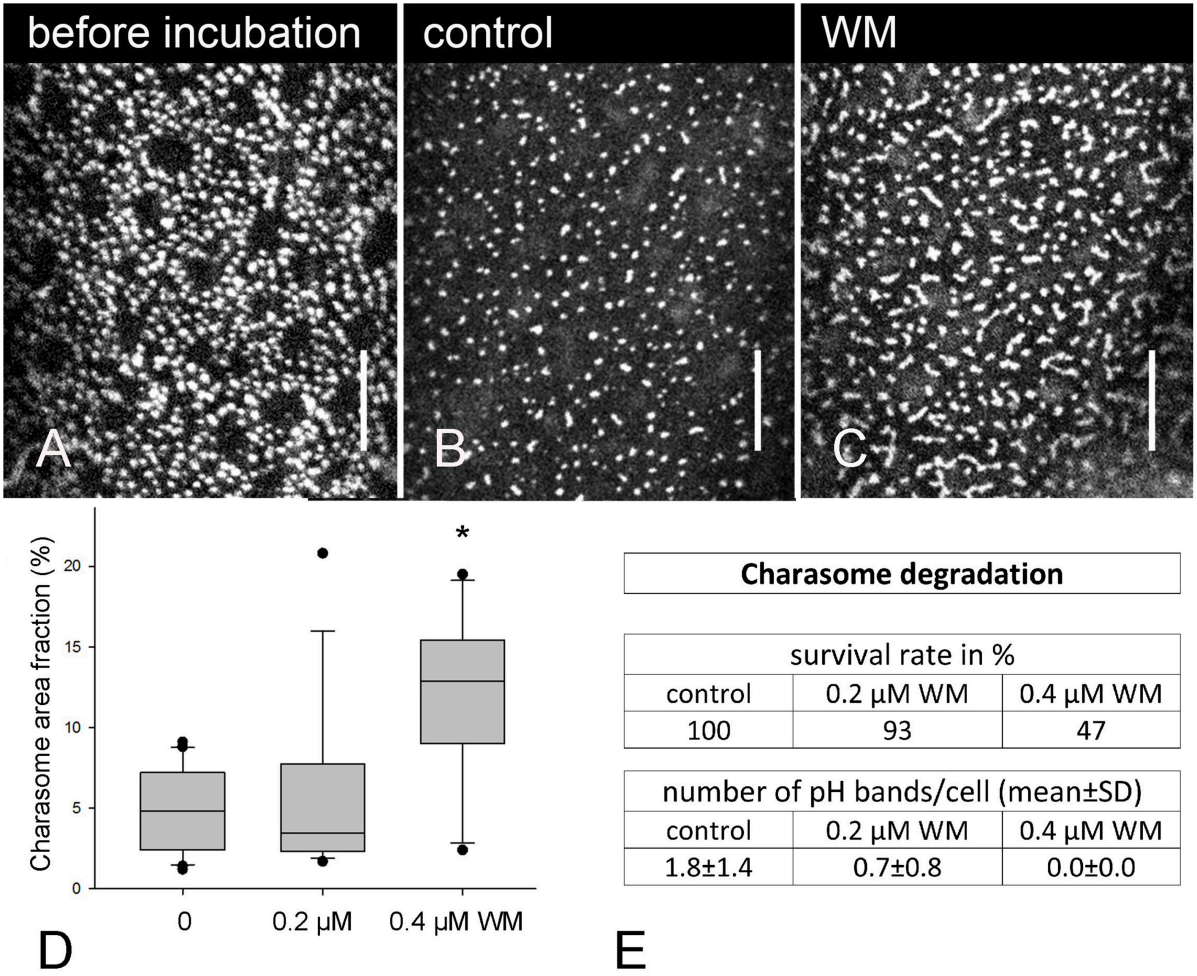
**Effect of wortmannin on charasome degradation in ***Chara*** internodal cells**. **(A)** AM1-44 stained charasomes at the acid region of a cell exposed to standard light/dark conditions for 8 days (before treatment). **(B)** AM1-44 stained charasomes in a DMSO-treated cell after 8 days incubation in darkness (control). **(C)** AM1-44 stained charasomes in a wortmannin (WM)-treated cell after 8 days incubation in darkness. **(D)** Maximum charasome area fractions in control and wortmannin-treated cells are compared in box-and-whisker plots. Shown are median values with upper and lower quartiles (boxes), whiskers indicating the 10th and 90th percentiles and outliers (dots). Values are based on data obtained from 12 to 16 cells. Differences between median values of control and 0.4 μM wortmannin treated cells are significant (asterisk; *P* = 0.003, Kruskal–Wallis one way analysis of variance on ranks). **(E)** Table showing the survival rate and pH banding activity of the cells analyzed in **(D)**. Bars are 10 μm.

For degradation studies, cells were isolated from the thallus and exposed to light (about 5 μE.m^−2^.s^−1^, 16/8 h light dark regime) for at least 1 week. These cells contained abundant charasomes similar to those illustrated in Figure 6A. Cells were then dark-incubated in various concentrations of wortmannin and in the corresponding amount of solvent (controls). After 8–13 days, charasomes were stained with FM/AM dyes and analyzed in the confocal laser scanning microscope, in order to determine the maximum charasome area fraction (% of cell surface area covered by charasomes) for each cell. Representative images for maximum charasome area fractions in a control cell and in a cell treated with 0.4 μM wortmannin are shown in Figures 6B,C. The statistical analysis confirmed a significant inhibitory effect of 0.4 μM wortmannin on charasome degradation (Figure 6D). Higher concentrations of wortmannin were lethal when applied for a longer time period, and even at 0.4 μM about 50% of the cells died (47% in the experiment illustrated in Figure 6E). Surviving cells, unlike the control cells and the cells treated with 0.2 μM wortmannin, did not develop acid and alkaline bands after 2 h exposure to light (Figure 6E). Although the ability to develop pH bands is not a prerequisite for charasome degradation (contrary to charasome formation; see below), the failure to do so may indicate a general, unspecific effect of wortmannin during long-time incubation, and the results therefore must be cautiously interpreted.

**Figure 7:**
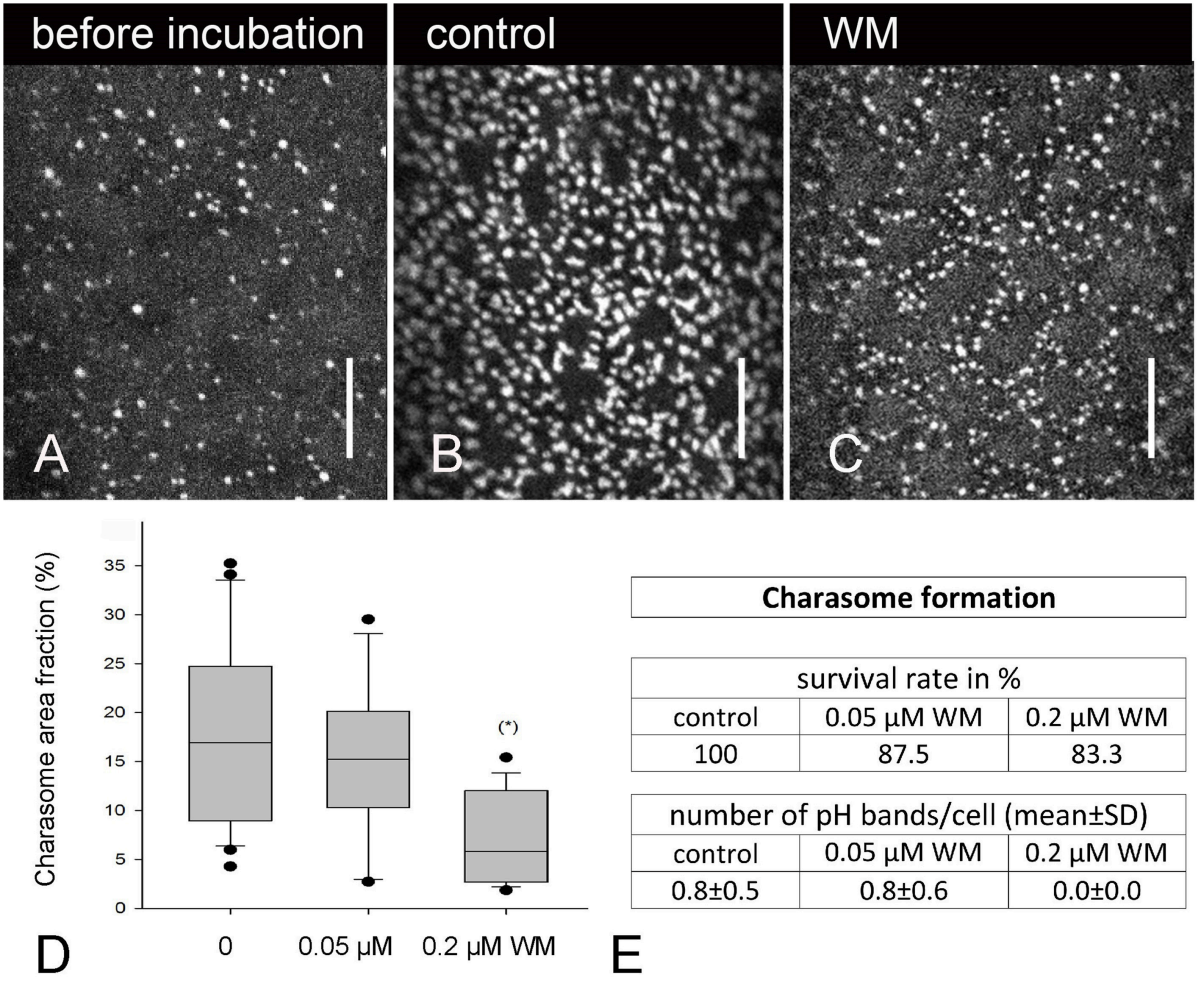
**Effect of wortmannin on charasome formation in ***Chara*** internodal cells**. **(A)** AM1-44 stained charasomes in a cell which was dark-incubated for 2 weeks. **(B)** AM1-44 stained charasomes at the acid region of a control cell exposed to standard light conditions for 8 days. **(C)** AM1-44 stained charasomes in a cell treated with 0.2 μM wortmannin (WM) under standard light conditions for 8 days. **(D)** Maximum charasome area fractions in control and in wortmannin-treated cells are compared in box-and-whisker plots. Shown are median values with upper and lower quartiles (boxes), whiskers indicating the 10th and 90th percentiles and outliers (dots). Values are based on data obtained from 15 to 17 cells. Differences between median values of control cells and cells treated with 0.2 μM wortmannin are significant (asterisk; *P* = 0.003, Kruskal–Wallis one way analysis of variance on ranks). **(E)** Table showing the survival rate and pH banding activity of the cells analyzed in **(D)**. Bars are 10 μm.

Charasome formation was studied in cells previously exposed to darkness for at least 10 days. These cells, which contained only few, small charasomes (Figure 7A), were then treated with solvent and wortmannin, respectively, and exposed to standard light-dark conditions for about 8 days. Significantly lower median values for charasome area fraction were obtained in cells treated with 0.2 μM wortmannin, in comparison to control cells, and to cells treated with lower concentrations (Figures 7B–D). Still, unlike charasome degradation, charasome formation strictly depends on the development of acid and alkaline bands (Schmoelzer et al., 2011), and Figure 7E shows that pH banding was significantly disturbed at 0.2 μM wortmannin. Therefore, the inhibitory effect of wortmannin on charasome development was probably due to inhibition of pH banding.

## Discussion

### The TGN plays a central role in wortmannin-induced fusion of MVBs in characean internodal cells

Wortmannin causes the accumulation and enlargement of organelles which can be stained with endocytic markers, e.g., FM/AM dyes. These wortmannin-induced compartments were found to consist mainly of enlarged, homotypically fused MVBs, but some enlarged MVBs also contained proteins characteristic for the TGN (“mixed compartments”; e.g., Lam et al., 2007a,b; Wang et al., 2009; Takáč et al., 2012). During the course of this study we observed that fusion of MVBs in wortmannin-treated characean internodal cells occurred only in clusters where MVBs were surrounded by and continuous with tubular membranes. Electron microscopy and double immunofluorescence showing co-localization of ARA7 and SCAMP1 epitopes at wortmannin compartments indicate that these tubules were of TGN origin. These findings, as well as images of MVBs with tubular extensions in untreated characean internodal cells, and in the green alga *Botryococcus* (Noguchi and Kakami, 1999), are consistent with the idea of TGN-to-MVB maturation (Scheuring et al., 2011). Similar tubules are present within the enlarged MVBs. Therefore, in characean internodal cells, MVBs do not only fuse with each other, but also with tubular components of the TGN. In many other plant cells, only TGN vesicles have been described to fuse with the MVBs (Takáč et al., 2012).

Mature branchlet internodal cells contain only very few MVBs in the endoplasm. Hence, the chance to meet and fuse is low, as long as cytoplasmic mass streaming is continuous, so that organelles keep distance to each other. TGNs are much more abundant in characean internodal cells. We therefore speculate that MVBs in wortmannin-treated cells gather by means of TGN tubules. Of course, the mechanism by which this occurs remains to be elucidated, but this would explain why fusing MVBs were only observed in tubule-containing clusters.

### Wortmannin induces reorganization of the TGN in *Chara* internodal cells

**Figure 8:**
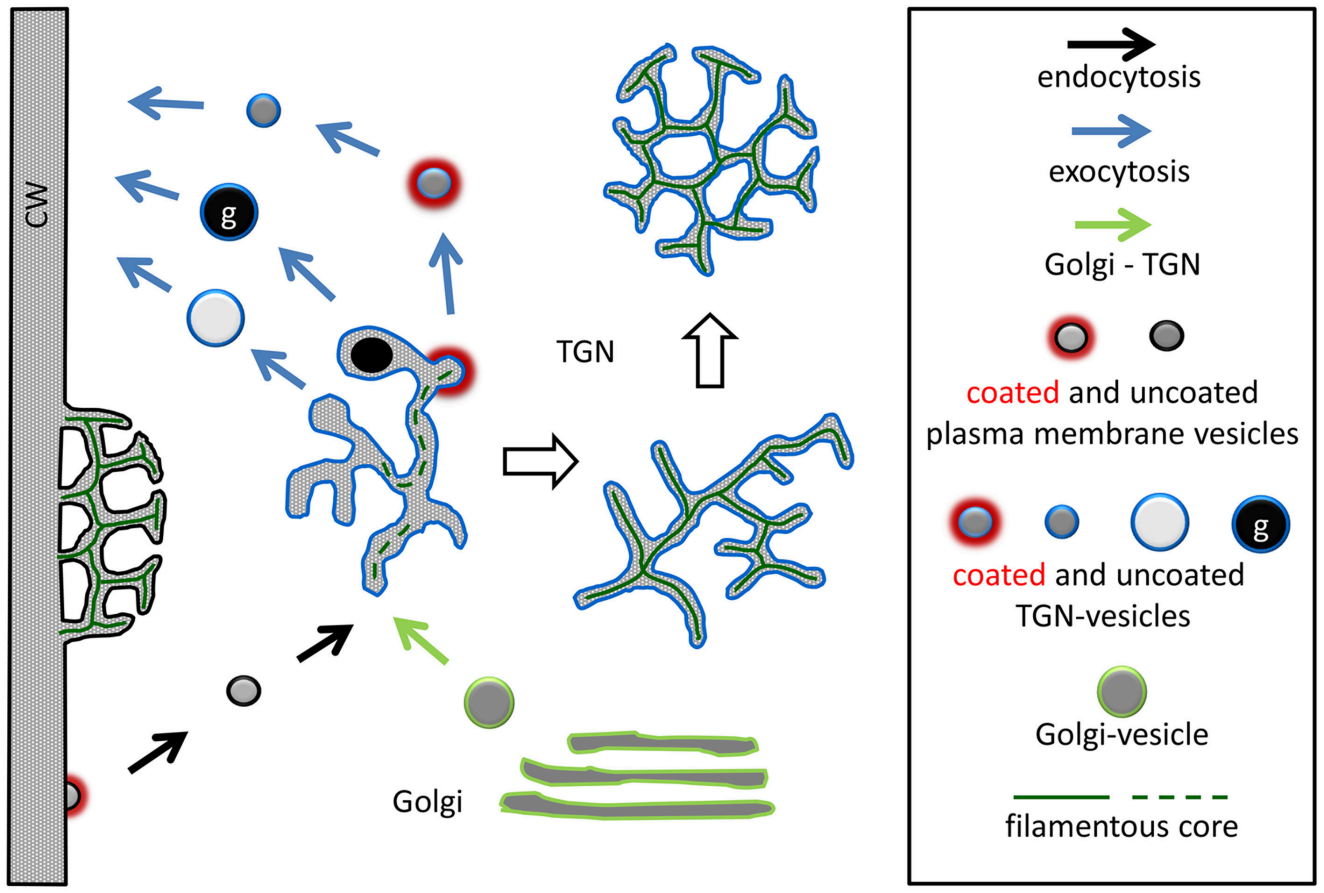
**Schematic summary showing the formation of a wortmannin-modified TGN and its similarity with a charasome**. The charasome at the left side of the image is neither growing, nor degrading, as suggested by the absence of coated pits. The periplasmic space of the charasome contains a filamentous core, and is continuous with the cell wall (CW). Treatment of cells with wortmannin neither arrests formation of endosomes (constitutive endocytosis) from smooth plasma membrane, nor production of Golgi vesicles, both required for TGN formation. The “normal” TGNs are involved in the production of various vesicles (including glycosomes, g) designed for exocytosis. A filamentous core is occasionally visible in TGN tubules and TGN-derived vesicles. Other TGNs (probably those equipped with a specific set of PI3Ps and/or PI4Ps) are much more affected by wortmannin. They lose the capacity to pinch off vesicles, eventually enlarge, and finally form compact charasome-like structures where a filamentous core is visible in all tubules. Only approximately drawn to scale.

The TGN is a highly variable organelle and its size and morphology undergo considerable variation during the cell cycle (Kiermayer, 1981; Noguchi and Kakami, 1999). Here we describe that under the influence of wortmannin, some TGNs in *Chara* internodal cells greatly increase their size, lose their coat and the capacity to pinch off vesicles, and, finally, form compact three-dimensional structures consisting of branching/anastomosing membranous tubules, which have a striking resemblance with charasomes (see Figure 8 for a schematic summary). To our knowledge, this is the first description of extensive wortmannin-induced reorganization of TGNs. In other plant cells, TGNs have been described to be depleted during wortmannin-treatment (Takáč et al., 2012), or to be wortmannin-insensitive (Jaillais et al., 2008).

The effects of wortmannin on cellular organelles are due to the inhibition of phosphoinositide kinases. At low concentrations (up to 1 μM) wortmannin has been reported to be a specific inhibitor of phosphoinositide 3 (PI3) kinases, but at higher concentrations other, PI3 kinase-related enzymes, and phosphatidylinositol 4 (PI4) kinases are also affected (Powis et al., 1994; Matsuoka et al., 1995; Krinke et al., 2007; Takáč et al., 2012 for references). Phosphatidylinositol kinases play an important role in cellular metabolism. They produce inositol phosphates which regulate vital cellular processes, including signal transduction and membrane trafficking. Phosphoinositides act as docking sites for proteins containing specific lipid-binding domains (van Leeuwen et al., 2004; Di Paolo and De Camilli, 2006; Krishnamoorthy et al., 2014). Different forms of phosphoinositides, regulated by the combined action of specific kinases and phosphatases, segregate on different intracellular membranes, thereby establishing an identity code of organelles involved in traffic, and enabling a fine tuned cargo delivery to the target membrane (Di Paolo and De Camilli, 2006; Balla, 2013; Sekeres et al., 2015). PI3P has been shown to be enriched in late endosomes and in the tonoplast (Kim et al., 2001; Vermeer et al., 2006; Krishnamoorthy et al., 2014), which is consistent with the effect of wortmannin on MVBs and vacuoles. In contrast, PI4P has been reported to localize preferentially in the plasma membrane and in the Golgi bodies (Vermeer et al., 2009), but also in post Golgi, TGN, and recycling endosomes, respectively (Kang et al., 2011; Simon et al., 2014; Sekeres et al., 2015). Mutants that decrease PI4P levels at the TGN were found to slow, or uncouple coat assembly via interference with clathrin adaptor proteins (Daboussi et al., 2012). Whether this is a direct or indirect effect (e.g., via reduced V-ATPase activity; Liu et al., 2016) remains to be investigated. In any case, it is reasonable to conclude that the disappearance of vesicles from the TGN of *Chara* internodal cells is due to impaired coat assembly, caused by wortmannin-induced inhibition of PI4 kinase(s) (compare Bednarek and Backues, 2010). In this context, it is interesting to note that epsin, an amphipatic membrane protein that drives the curvature of the coated pits in conjunction with clathrin polymerization, has an absolute requirement for PI(4,5)P_2_ (Ford et al., 2002; Boucrot et al., 2012). Another likely candidate for the observed changes is the small GTPase, RabA1d, which regulates vesicular trafficking at TGN, and was identified as a new protein negatively affected by wortmannin (Takáč et al., 2012). These known effects of wortmannin suggest that the TGNs in wortmannin-treated *Chara* internodal cells increase their size because of the failure to release vesicles via a PI4 kinase dependent and clathrin-mediated mechanism, and because of the ongoing supply of endocytic vesicles from the plasma membrane (see below). We speculate that this causes the accumulation of a protein involved in membrane curvature, which finally leads to the compactification of the organelle. Interestingly, however, TGN tubules in root cells of *Arabidopsis* PI4 kinase mutants appear to be blown up, but are still equipped with clathrin coats (Kang et al., 2011).

During the course of this study, we found a profound effect of wortmannin on the morphology of the TGNs in *Chara* internodal cells. However, not all TGNs were similarly affected. TGNs with normal size and morphology were always present in wortmannin-treated cells, even when they contained enlarged, vesicle-free, or compact TGNs. Therefore, the TGNs in wortmannin-treated *Chara* internodal cells can be present in three different forms, (1) as normal organelles (eventually enlarged), (2) as vesicle-free, loose, or compact meshworks of uncoated tubules, and (3) as a component of mixed compartments together with MVBs. These findings may reflect the existence of different populations or “maturation stages” of TGNs, as described in *Arabidopsis* root and hypocotyl cells (e.g., Viotti et al., 2010; Kang et al., 2011; Uemura et al., 2014) and in yeast (Mogelsvang et al., 2003). It is possible that these populations differ in the PI3P/PI4P content of their membranes, which could explain the multiple effects of wortmannin.

Several authors have reported that treatment of cells with wortmannin is irreversible (e.g., Powis et al., 1994). In characean internodal cells, we found that the partial inhibition of cytoplasmic streaming and pH banding, as well as the formation of wortmannin-induced compartments, were reversible. The recovery took, however, several days, consistent with the strong covalent binding of wortmannin to the ATP-binding site of its target enzyme (Wymann et al., 1996; Walker et al., 2000; Yuan et al., 2007). This binding possibly also explains our observations that the size of wortmannin compartments (TGN as well as MVBs) transiently increased during recovery from treatment with 25 μM wortmannin. Part of the recovery time can thus be considered as prolonged drug treatment.

### Are similar mechanisms involved in the formation of charasomes and wortmannin–modified TGNs?

A characteristic feature of charasome tubules is a central core, which either appears as a filament, or as a row of dots, and which is surrounded by granular or fibrous material (Franceschi and Lucas, 1980). The granular or fibrous material is visible only after fixation with tannic acid-containing glutaraldehyde (Franceschi and Lucas, 1980). A central core (filamentous or row of dots) with a similar diameter is also seen in some, but not all regions of the TGN and in TGN-derived vesicles in untreated *Chara* internodal cells. In the non-coated, wortmannin-modified TGNs, the central core was always present. This finding possibly indicates that this structure is involved in the formation of membranous tubular meshworks, although its nature is completely unknown so far. It also does not correspond to the proteoglycan described in various studies to be present in the periplasmic space of charasome tubules (Franceschi and Lucas, 1981a; Beljanski et al., 1995; see above). In addition, membrane-localized proteins are likely to play a role in sensing and establishing membrane curvature, like BAR-domain-containing proteins, e.g., arfaptins, amphiphysin, or endophilin, which bind to the TGN membranes in a PI4P-dependent manner (Shin et al., 2012; Cruz-Garcia et al., 2013), and which are remarkably effective in promoting membrane tubulation (Peter et al., 2004; Boucrot et al., 2012; Cruz-Garcia et al., 2013; Simunovic et al., 2013). Future work will show whether these or similar proteins are involved in the formation of charasomes and/or wortmannin-modified TGNs. In this respect, it is also important to note ARA6, an unconventional RAB5 GTPase residing in the plasma membrane, including charasomes, and in the TGN of *Chara* internodal cells (Hoepflinger et al., 2013). The exact function of this small GTPase is unknown so far, but it is likely involved in endocytosis and/or exocytosis (Ebine et al., 2012).

Scanning electron images suggest that during their development, charasomes undergo a transition from a loose network of long tubules to a complex anastomosing network of short tubules (Chau et al., 1994). The charasome tubules grow by fusion of TGN-derived vesicles with the plasma membrane, in the absence of membrane recycling (endocytosis; Franceschi and Lucas, 1980, 1982; Pesacreta and Lucas, 1984). Coated pits are abundant at the cytoplasmic surface of growing charasomes, and appear to fuse with each other, thereby forming the charasome tubules (Lucas and Franceschi, 1981). The cytoplasmic membrane (the inner surface) of charasomes in mature, untreated cells, however, is mostly uncoated (Franceschi and Lucas, 1980; Sommer et al., 2015). A similar sequence of events can be observed during formation of wortmannin-modified TGNs. Loose meshworks with clathrin-coated regions develop into compact, three-dimensional meshworks, which are devoid of a clathrin coat. The effect of wortmannin on the morphology of the TGNs in *Chara* internodal cells is likely to be due to the inhibition of PI3 and/or PI4 kinases, which probably leads to a depletion of phosphoinositides by disturbing the fine balance existing between PI kinases and PI phosphatases, and to the inhibition of coat assembly required for the release of vesicles (see above). We therefore speculate that similar mechanisms and molecules are involved in formation of charasomes.

### Clathrin-dependent constitutive and wounding-induced endocytosis are not arrested by wortmannin

It has been reported that wortmannin inhibits endocytosis (Bandmann and Homann, 2012; Ito et al., 2012 for references). In *Chara* internodal cells, wortmannin did not arrest the internalization of FM/AM dyes up to a concentration of 50 μM. FM/AM internalization in characean internodal cells has been shown to be active, and fluorescent organelles co-labeled with markers for the TGN and for MVBs, suggesting that these dyes can be used as endosomal markers (Klima and Foissner, 2008; Hoepflinger et al., 2013). In wortmannin-treated cells the number of FM/AM-stained endosomes was significantly lower as compared with the controls, but this is at least partly due to the formation of wortmannin-induced aggregates and ring-like compartments. In addition, electron micrographs show not only coated pits, but also coated vesicles at regions with smooth plasma membrane, i.e., between charasomes, which confirms that constitutive, clathrin-dependent endocytosis is not arrested by wortmannin in *Chara* internodal cells.

The deposition of a cellulosic wall beneath healing puncture wounds requires fusion of secretory vesicles with the plasma membrane and membrane recycling via coated vesicles, whereas the deposition of material in response to local irradiation occurs in the absence of membrane recycling (Klima and Foissner, 2011; Foissner and Wasteneys, 2012). Surprisingly, wortmannin neither had a detectable effect on healing of wounds induced by local irradiation (accumulation and fusion of organelles in the absence of membrane recycling), nor disturbed the healing of puncture wounds (fusion of vesicles with the plasma membrane and membrane recycling). This is in line with electron microscopical images showing that, even in cells treated with 50 μM wortmannin for 2 h, secretory vesicles required for wound healing were still present in sufficient number, and that wortmannin had only a small effect on clathrin-dependent plasma membrane retrieval. Wortmannin has therefore different effects on the release of coated vesicles from the plasma membrane and from (some of) the TGNs, respectively. This finding may reflect different membrane compositions regarding the type and amount of specific phosphoinositides (see above). Our data also show that wortmannin neither prevented local reorganization of actin cytoskeleton, nor significantly inhibited acto-myosin dependent transport, both required for delivery of secretory vesicles and other organelles toward the wound (Foissner and Wasteneys, 2012). This is consistent with our finding, that acto-myosin-dependent mass streaming of the endoplasm continued in the presence of 25 and 50 μM wortmannin for up to 2.5 h, although at lower rates, which may be due to the inhibition of a myosin light chain kinase (e.g., Nakanishi et al., 1992; Powis et al., 1994; Burdyga and Wray, 1998). In the mesophyll of *Lemna*, wortmannin inhibited actin-dependent reorientation of chloroplasts at much lower concentrations than those used in our study, but this effect is likely to be due to the interruption of a light-induced signaling cascade (Grabalska and Malec, 2004).

### Effect of wortmannin on charasome degradation and formation

The formation of wortmannin-induced compartments was studied with concentrations of 25 and 50 μM, which were lethal when applied for several days. Since the study of charasome formation and degradation requires a treatment time of at least 1 week, we had to use much lower concentrations (between 0.05 and 0.4 μM).

When *Chara* internodal cells are exposed to darkness, charasomes degrade via clathrin-dependent endocytosis (own, unpublished results). We are aware, that long-term treatment of cells with inhibitors must be cautiously interpreted because of unspecific side effects, and because of the rather low concentrations used, which had no significant effect on the size of FM/AM-stained organelles (data not shown). However, we assume that at these low concentrations wortmannin affects at least the activity of PI3 kinases (see Section Introduction) and retards the degradation of charasomes via an inhibitory effect on clathrin-dependent membrane retrieval. Charasome degradation was not totally inhibited, and this adds further support to the hypothesis that in *Chara*, plasma membrane recycling (at charasomes and at smooth plasma membrane regions) is less affected by wortmannin, than the release of coated vesicles from (at least some of) the TGNs.

So far, little can be said about the effect of wortmannin on charasome formation. Charasome formation is strictly dependent on the pH banding activity, which in turn requires photosynthesis and cytoplasmic streaming (e.g., Bulychev et al., 2001; Schmoelzer et al., 2011). The pH banding activity was significantly inhibited after long-time treatment with 0.2 μM wortmannin and this is probably the main reason why these cells contained significantly lower charasome area fractions.

## Author contributions

IF acquired funding and conceived the study. IF, AS, MH, and MCH designed the research; IF, AS, MH, and MA performed the research; AS, MH, MCH, and MA analyzed the data; IF drafted the manuscript; IF, AS, MH, MCH, and MA contributed to the final manuscript. All authors approved the final version of the manuscript.

### Conflict of interest statement

The authors declare that the research was conducted in the absence of any commercial or financial relationships that could be construed as a potential conflict of interest.

## Supplementary material

Supplementary Video 1 **Cortex of a DMSO-treated, AM4-65-stained *Chara* internodal cell**. Only delicate mobile organelles are occasionally seen between charasomes (encircled area). Interval between consecutive frames is 3.7 s; playback rate of movie is 5 frames per second.

Supplementary Video 2 **Cortex of a *Chara* internodal cell treated with 50 μM wortmannin for 2 h and stained with AM4-65**. The starting point of a large mobile organelle is indicated by an arrow, immobile organelles are charasomes. Interval between consecutive frames is 1 s; playback rate of movie is 5 frames per second.

Supplementary Figure 1 **Schematic longitudinal section through a characean internodal cell**. In the genus *Chara*, the plasma membrane (red line) adjacent to the cell wall (CW) may form convoluted domains (charasomes, arrows). Subcortical actin filament bundles (black lines) are present along the inner side of the cortical, stationary chloroplasts (C). Nuclei (N), Golgi bodies (G), *trans*-Golgi networks (TGNs), and multivesicular bodies (MVB) are predominantly located in the streaming endoplasm. Mitochondria (M), and various vesicles (v) may shuttle between cortex and endoplasm. V, small vacuole; cV, central vacuole. Organelles relevant for this study are shown in red. Only approximately drawn to scale.
