# Supplementary figures and images for: Is Wortmannin-Induced Reorganization of the trans-Golgi Network the Key to Explain Charasome Formation?
Source: Front Plant Sci. 2016 Jun 3;7:756. doi: 10.3389/fpls.2016.00756 (PMC4891338; doi:10.3389/fpls.2016.00756)

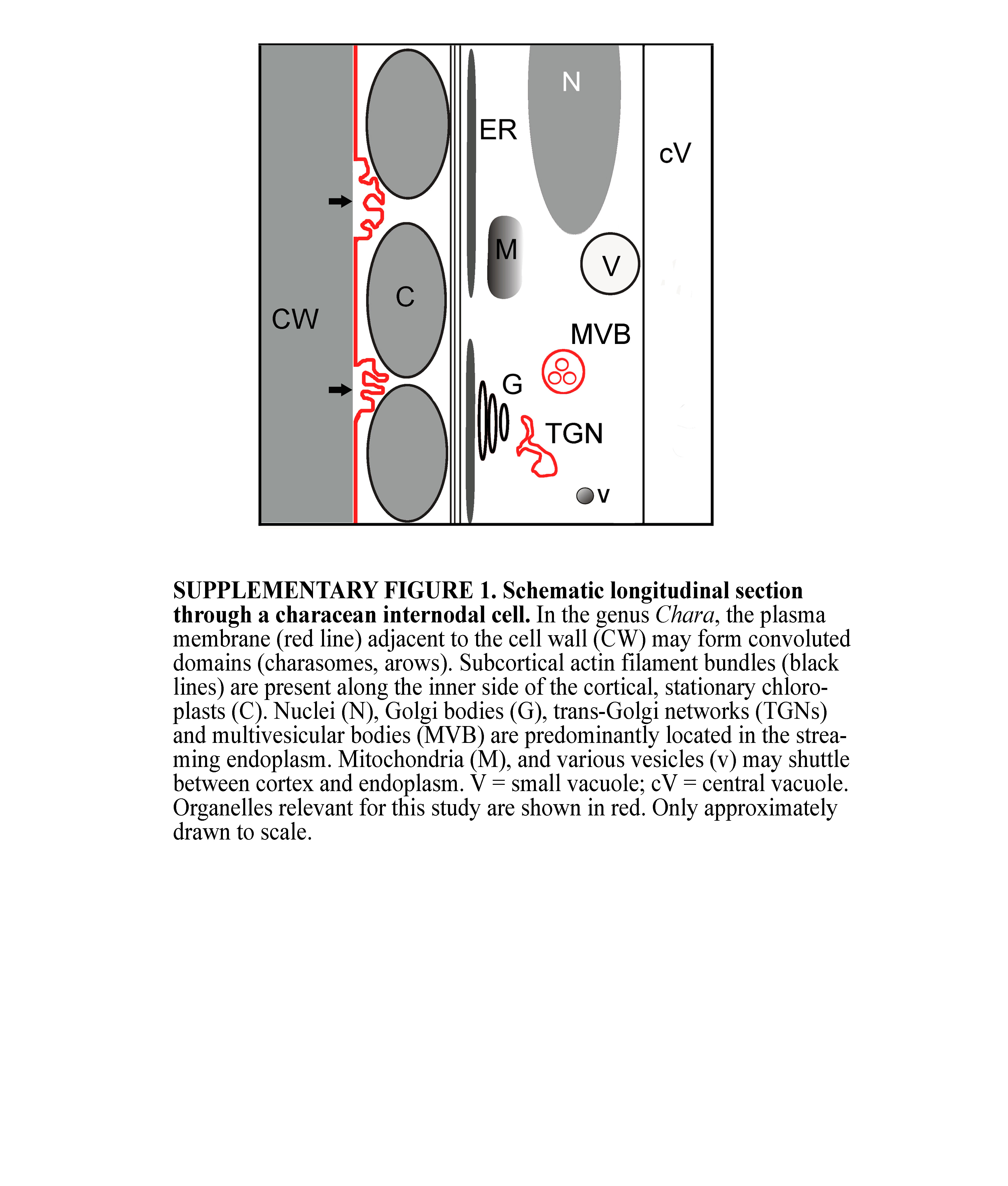

Supplement: Supplementary Figure 1 — Schematic longitudinal section through a characean internodal cell. In the genus Chara, the plasma membrane (red line) adjacent to the cell wall (CW) may form convoluted domains (charasomes, arrows). Subcortical actin filament bundles (black lines) are present along the inner side of the cortical, stationary chloroplasts (C). Nuclei (N), Golgi bodies (G), trans-Golgi networks (TGNs), and multivesicular bodies (MVB) are predominantly located in the streaming endoplasm. Mitochondria (M), and various vesicles (v) may shuttle between cortex and endoplasm. V, small vacuole; cV, central vacuole. Organelles relevant for this study are shown in red. Only approximately drawn to scale. [file Image1.jpg]
